# Surfactant Protein D Modulates HIV Infection of Both T-Cells and Dendritic Cells

**DOI:** 10.1371/journal.pone.0059047

**Published:** 2013-03-18

**Authors:** Jens Madsen, Gaurav D. Gaiha, Nades Palaniyar, Tao Dong, Daniel A. Mitchell, Howard W. Clark

**Affiliations:** 1 Department of Child Health, Sir Henry Wellcome Laboratories, Clinical and Experimental Sciences, Faculty of Medicine, University of Southampton, Southampton General Hospital, Southampton, United Kingdom; 2 Institute for Life Sciences, University of Southampton, Southampton, United Kingdom; 3 Ragon Institute of Massachusetts General Hospital, Massachusetts Institute of Technology and Harvard, Charlestown, Massachusetts, United States of America; 4 SickKids Research Institute, University of Toronto, Toronto, Canada; 5 Medical Research Council Human Immunology Unit, Weatherall Institute of Molecular Medicine, University of Oxford, Oxford, United Kingdom; 6 Clinical Sciences Research Institute, Warwick Medical School, University of Warwick, Coventry, United Kingdom; New York University, United States of America

## Abstract

Surfactant Protein D (SP-D) is an oligomerized C-type lectin molecule with immunomodulatory properties and involvement in lung surfactant homeostasis in the respiratory tract. SP-D binds to the enveloped viruses, influenza A virus and respiratory syncytial virus and inhibits their replication *in vitro* and *in vivo*. SP-D has been shown to bind to HIV via the HIV envelope protein gp120 and inhibit infectivity *in vitro*. Here we show that SP-D binds to different strains of HIV (BaL and IIIB) and the binding occurs at both pH 7.4 and 5.0 resembling physiological relevant pH values found in the body and the female urogenital tract, respectively. The binding of SP-D to HIV particles and gp120 was inhibited by the presence of several hexoses with mannose found to be the strongest inhibitor. Competition studies showed that soluble CD4 and CVN did not interfere with the interaction between SP-D and gp120. However, soluble recombinant DC-SIGN was shown to inhibit the binding between SP-D and gp120. SP-D agglutinated HIV and gp120 in a calcium dependent manner. SP-D inhibited the infectivity of HIV strains at both pH values of 7.4 and 5.0 in a concentration dependent manner. The inhibition of the infectivity was abolished by the presence of mannose. SP-D enhanced the binding of HIV to immature monocyte derived dendritic cells (iMDDCs) and was also found to enhance HIV capture and transfer to the T-cell like line PM1. These results suggest that SP-D can bind to and inhibit direct infection of T-cells by HIV but also enhance the transfer of infectious HIV particles from DCs to T-cells *in vivo*.

## Introduction

Surfactant protein D (SP-D) is a secreted soluble C-type lectin with a collagenous domain belonging to the group termed collectins [1]. Collectins are comprised of structural units composed of three polypeptide chains. Each chain has four domains: 1) a N-terminal region with cysteine residues involved in the higher oligomerisation of the mature SP-D molecule; 2) a collagen like region with hydroxylated lysine and proline residues; 3) an a-helical coiled-coil neck region where the initial trimeric unit formation is initiated and 4) a calcium-dependent carbohydrate recognition domain (CRD) at the C-terminus that is conserved across species [2]. The mature SP-D protein is oligomerized into a dodecameric molecule with four trimeric units that come together involving the N-terminal region with two cysteine residues in each poly peptidechain [3], [4]. This collectin family includes other members such as surfactant protein A (SP-A) and mannose binding lectin (MBL). The affinity of a single CRD towards a single carbohydrate epitope found of the surface of a pathogenic microorganism is low. However, through the formation of the trimeric unit and the additional higher order oligmerization of the mature molecule, collectins have a high avidity for repeated carbohydrate structures as seen on micro organisms. SP-D promotes agglutination and phagocytosis of micro organisms [5], [6], has a chemotactic effect on phagocytes [7], [8], modulates inflammatory responses [6], [9], [10], has a direct anti-microbial effect [11], [12] and has been shown in *in vivo* models to be involved in the response to and clearance of viruses such as influenza A virus (IAV) and respiratory syncytial virus (RSV) [13]–[15].

Human immunodeficiency virus (HIV) is the causative agent of Acquired Immune Deficiency Syndrome (AIDS). According to the World Health Organisation (WHO) 34 million people were living with HIV, 2.7 million people were newly infected and 1.8 million died of AIDS in 2010 [16]. The envelope protein (Env) is a trimer made of three copies of each glycoprotein 120 (gp120) and glycoprotein 41 (gp41). Both proteins play crucial roles in the entry process of the virus into cells by mediating the fusion between viral and cellular membranes during the entry process (recently reviewed by Caffrey) [17]. Gp120 is highly glycosylated with approximately half of the molecular mass composed of N-linked glycans [18]. This makes a “glycan shield” that masks HIV from host immune recognition [19], contributes to correct folding of the protein and helps virions bind to the host cell surface [20].

SP-D was originally isolated from lung surfactant but has been shown to be expressed on all mucosal surfaces including gastrointestinal and female genitourinary tracts [21]–[23]. SP-D can be isolated from various body fluids including amniotic fluid, bronchoalveolar lavage (BAL), saliva and tear fluid [22], [24], [25]. Direct interaction between MBL and HIV has been shown by several groups [26], [27] and we have recently shown that SP-A binds to HIV and inhibits infection of CD4+ cells but enhances dendritic cell-mediated viral transfer to CD4+ cells [28]. SP-D has also been shown to interact with HIV - specifically gp120 - and the interaction was dependent on the degree of glycosylation of gp120 [29]. Furthermore, SP-D was found to inhibit HIV infection of the U937 monocyte-like cell line [29]. Here we show that SP-D binds to several strains of inactivated HIV particles and SP-D specifically interacts with gp120 at both pH 7.4 and pH 5.0. The SP-D - gp120 interaction was investigated using ELISA assays and surface plasmon resonance. The interaction was further characterized using inhibition assays with different hexoses and the epitope that SP-D interacts with on gp120 was mapped using three gp120 binding proteins. SP-D agglutinates HIV particles and gp120 molecules in the presence of calcium and the addition of EDTA dissociated the agglutinated complexes. SP-D enhanced the binding of HIV to immature monocyte derived dendritic cells (iMDDCs). SP-D also enhanced the transfer of HIV from iMDDCs to CD4+ T-cells at pH values of 7.4 and 5.0. Thus, SP-D appears to be a dual modulator of HIV infection by protecting CD4+ T-cells from direct infection but enhancing the transfer of HIV from DCs to CD4+ T-cells.

## Materials and Methods

### Ethics Statement

Human bronchoalveolar lavage (BAL) was obtained from patients diagnosed with pulmonary alveolar proteinosis undergoing this procedure for therapeutic purposes. The procedure was approved by the London National Health Service Research Ethics Committee and BAL was donated by informed patients with written consent. Blood was donated by informed male participants (ages: 18–40 years) with written consent and the procedure was approved by the University Research Ethics Committee at University of Oxford.

### Human immunodeficiency virus and T cell lines

Aldrithiol-2 (AT-2) inactivated HIV BaL particles were provided by Mr. Julian Bess of the National Cancer Institute AIDS Vaccine Program (SAIC,Frederick, MD). Infectious HIV BaL (ARP118) and HIV IIIB (ARP101.1) were obtained from the National Institute of Biological Standards and Control (NIBSC) AIDS Reagent program. High titer stocks were generated by infecting 1×10^7^ pelleted PM1 cells for 90 min at 37 °C/5% CO2 with 500 µL of viral supernatant. After incubation, 10 mLs of RPMI 1640 supplemented with 50 U/ml penicillin/streptomycin (Life Technologies), 2 mM L-Glutamine (Sigma-Aldrich), and 10% (v/v) foetal bovine serum (FBS) (Sigma-Aldrich) (R10) was added and the virally infected cell culture was transferred to a T25 flask for growth. Aliquots (1 mL) were taken on days 5 and 7 and assayed for Reverse Transcriptase activity using the SPA Quant-T-RT assay kit (Amersham) according to the manufacturer's instructions.

The PM1 (ARP057) cell line and the C8166 (ARP013) cell line were obtained from the NIBSC AIDS Reagent Program. PM1 is a sub clone of the neoplastic T-cell line Hut78 [30] and C8166 is an immortalized human umbilical cord blood lymphocyte cell line developed by cocultivation or fusion of fresh cells with T cells cultured from leukemia-lymphoma patients containing human T-cell leukemia-lymphoma virus [31].The cells were grown in R10 and split routinely 1∶8 to maintain the cells in the exponential growth phase. Cells were routinely tested for mycoplasma infection.

### Proteins

SP-D was purified by sugar-affinity chromatography from therapeutic bronchoalveolar lavage (BAL) obtained from patients diagnosed with pulmonary alveolar proteinosis as described previously [32]. MBL was purified from a pool of serum as described previously [27], [33]. Recombinant soluble DC-SIGN was produced as described previously [34]. The concentration of SP-D, MBL or DC-SIGN was determined by analysis of amino acid composition. Recombinant gp120 IIIB produced in Chinese hamster ovary (CHO) cells, biotinylated gp120 IIIB, and FITC-labeled gp120 IIIB were purchased from Immunodiagnostics. Cyanovirin (CVN) and polyclonal rabbit anti-CVN Ab were provided by Dr. Kirk Gustafson (National Cancer Institute, Frederick, MD). SP-D and MBL used in all cell-based assays were treated for endotoxin removal, by passing the protein solutions through a 10 mL Polymyxin B column (Detoxi-Gel; Pierce) in sterile PBS (pH 7.4). Remaining levels of endotoxin were assayed using a Limulus Amoebocyte Lysate kit, according to the manufacturer's instructions (Bio-Whittaker). An endotoxin level of <10 pg/µg of protein was judged acceptable for use in cell-based assays.

### Enzyme Linked Immunsorbent Assays (ELISAs)

Inactivated HIV BaL (10 µg/mL), gp120 IIIB (2 µg/mL), SP-D or MBL (2 µg/mL) was immobilized on 96-well plates (Nunc, Maxisorp) in 0.1 M sodium bicarbonate buffer (pH 9.6) at 4 °C for 18 h. The wells were washed with PBS and 0.05% (v/v) Tween 20 (PBST) and blocked in 3% (v/v) BSA for 2 h at 37 °C. Immobilization of virus was verified by incubation with a purified pooled anti-HIV Env IgG (kindly provide by Dr Quentin Sattentau (Dunn School of Pathology, Oxford University, Oxford, UK)), followed by peroxidase conjugated anti-human IgG, and detection using the H_2_O_2_-tetramethylbenzidine-based (H_2_O_2_-TMB) chromogenic substrate according to the manufacturer's instructions (Bio-Rad). After washing away excess BSA with PBST, the wells were incubated with increasing concentrations of SP-D (0–4 µg/mL) or AT-2 inactivated HIV BaL (0–10 µg/mL) in Tris buffered saline with calcium (TBSC: 20 mM Tris-HCl, 150 mM NaCl, 5 mM CaCl_2_, pH 7.4) with 25% (v/v) human serum or Tris buffered saline with EDTA (TBSE; 20 mM Tris-HCl, 150 mM NaCl, 2 mM EDTA, pH 7.4) with 25% (v/v) human serum. For binding experiments performed at a pH of 5.0, SP-D or AT-2 inactivated HIV BaL was incubated in 20 mM Sodium Acetate, 150 mM NaCl, 25% (v/v) human serum and either 5 mM CaCl_2_ or 2 mM EDTA. The wells were washed in the corresponding binding buffer, and incubated with biotinylated anti-human SP-D Ab against recombinant fragment of SP-D (1 µg/mL) at 37 °C for 2 h. Bound HIV particles were lysed in PBS with 1% (v/v) Triton X-100 and p24 Ag was measured by ELISA according to the manufacturer's instructions (Immunodiagnostics). The collectin-antibody complexes or p24 Ag were detected using HRP-streptavidin and H_2_O_2_-TMB. The absorbance (450 nm) of individual wells was measured by a spectrophotomer (Multiscan Ascent, Labsystems; Fisher).

### Dot Blot Assay

Purified human SP-D (100 ng), and recombinant gp120 (200 ng and 2-fold serial dilutions) were carefully dotted onto Hybond-C Extra nitrocellulose membrane discs (Amersham Biosciences) and allowed to dry in a Petri dish. Membranes were incubated with 2% (w/v) BSA in PBST for 2 h at room temperature. Membranes were washed three times with either 20 mL of TBSC or TBSE. Membranes were then incubated with 10 mL of TBSC or TBSE with SP-D (200 ng/mL) for 1 h. The membranes were washed as above, and incubated further with 10 mL of biotinylated rabbit polyclonal anti-human SP-D for 1 h. This antibody has previously been used for detection of SP-D [35], [36]. Unbound antibody was washed away as described above, and the membranes were incubated with HRP-streptavidin (1∶200) for 20 min. SP-D complexes were detected by ECL reagents (Amersham Biosciences).

### Surface Plasmon Resonance

SP-D (1 mg/mL) was immobilized on a flow cell at the approximate reading of 1000 resonance units, on a SA chip in a pH 5.0 Sodium Acetate buffer according to the manufacturer's instructions (Biacore). To determine whether HIV could bind to the immobilized SP-D, AT-2 inactivated HIV BaL particles (100 µg/mL) were passed over the cell in 10 mM HEPES (pH 7.4), 150 mM NaCl, 5 mM CaCl_2_, 0.005% (v/v) surfactant P-20, 0.02% (w/v) NaN_3_ (HSC) buffer or 10 mM HEPES (pH 7.4), 150 mM NaCl, 1 mM EDTA, 0.005% (v/v) surfactant P-20, 0.02% (w/v) NaN_3_ (HSE) at 10 µL/min for 2 min at 25 °C. The complexes were allowed to disassociate for 90 sec, and bound viral particles were removed with two 20 µL washes of HSE and the chip was re-equilibrated with 20 µL of HSC buffer. Competition assays using the SP-D chip were accomplished by flowing AT-2 inactivated HIV particles (100 µg/mL) in the presence of different concentrations of hexoses (maltose, mannose, GlcNac, galactose; 0–500 mM). The surface plasmon resonance response obtained in the absence of any competitor was considered as 100%, and the relative binding was calculated for each competitor concentration. To examine the effects of pH on the binding of HIV to immobilized SP-D, AT-2 inactivated HIV BaL particles (100 µg/mL) were diluted into a 50 mM Sodium Acetate, 50 mM Bis-Tris, 50 mM Tris, 150 mM NaCl, 5 mM CaCl_2_ buffer (Buffering capacity: pH 4.5–8.0) at various pHs (5.0, 5.5, 6.0, 6.5, 7.4). AT-2 inactivated HIV BaL particles (100 µg/mL) were passed over the flow cell in HSC at a particular pH at 10 µL/min for 2 min at 25 °C. The complexes were allowed to disassociate for 90 sec and bound viral particles were removed with two 20 µL washes of HSE and the chip was re-equilibrated with 20 µL of HSC buffer.

Association and dissociation rate constants of SP-D at various pHs were calculated by nonlinear fitting of the primary sensogram data using the BIAevaluation 2.0 software (Pharmacia Biosensor) and the method described by MacKenzie et al [37]. Relative affinity was calculated using these rate constants.

### Agglutination Assay

Agglutination assays were performed as described previously [38] by measuring the change in protein absorbance at 400 nm in a Cecil CE 292 spectrophotometer. Absorbance at 400 nm of 20 mM Tris-HCl (pH 7.4), 150 mM NaCl was stabilized for 3 min at 23 °C, at which SP-D (10 µg/mL), HIV MN (10 µg/mL), or gp120 IIIB (10 µg/mL) was added. After an additional equilibration for 3 min, CaCl_2_ (10 mM) was added to the sample cuvette and the absorbance was monitored at 30 sec intervals over 10 min. Reversal of virus and protein aggregation was achieved by addition of EDTA (10 mM) and absorbance at 400 nm was recorded for an additional 2 min.

### HIV infectivity assay

PM1 cells for HIV BaL and C8166 cells for HIV IIIB were infected with 3–log10 TCID50/mL of virus inoculum (HIV BaL or IIIB) that had been pre-incubated with 10 µL of serially diluted SP-D (final concentration: 1–10 µg/mL) for 1 h at 37 °C. 90 min post infection, the cells were washed extensively, resuspended in fresh R10, and aliquoted into 200 µL volumes containing 5×10^4^ cells/well. Infected cells were then incubated for 4–5 days before measuring extracellular p24 content from culture supernatants by ELISA (Immunodiagnostics).

### Competition assays

SP-D (0–40 µg/mL) was incubated simultaneously with the protein competitors recombinant soluble DC-SIGN, recombinant CD4 (both biotinylated) or the small lectin cyanovirin (CVN) in wells coated with recombinant gp120 IIIB (2 µg/mL). Bound biotinylated CD4 and DC-SIGN was detected by streptavidin-HRP incubation for 30 min at 37 °C, followed by H_2_O_2_-TMB.

### Generation and characterization of primary dendritic cells

Immature monocyte-derived DCs (iMDDCs) were generated from a highly enriched population of CD14 monocytes. Peripheral blood mononuclear cells (PBMCs) were isolated from blood obtained from healthy male donors (ages: 18–40) using a Ficoll-Hypaque density gradient (Amersham Biosciences). PBMCs were washed thoroughly with sterile PBS and contaminating red blood cells were lysed using ACK lysis buffer. PBMCs were labeled with anti-CD14 microbeads (Miltenyi Biotec) and CD14 cells were isolated using the AutoMACS (Miltenyi Biotec) magnetic cell sorter. The purity of the isolated CD14 cells was >95% as assessed by flow cytometry. iMDDCs were cultured from monocytes in the presence of IL-4 (500 U/mL; R&D Systems) and GM-CSF (800 U/mL; R&D Systems), with fresh cytokines added on days 3 and 5. At day 7, the phenotype of the cultured iMDDCs was confirmed by high CD11c and HLA-DR expression, and low CD83 expression as determined by flow cytometry (supplementary Figure S1).

### DC-gp120 binding assay

FITC-labeled gp120 IIIB was incubated in the presence of SP-D (5 µg/mL) in R10 made 5 mM CaCl_2_ for 1 h at 4 °C. SP-D-gp120 complexes were incubated with iMDDCs (1×10^5^) and incubated for 2 h at 4 °C to prevent uptake of gp120. Trypan blue exclusion staining revealed more than 95% viability after incubation with gp120 and SP-D. Cells were pelleted and washed in PBS with calcium and magnesium and then fixed in 3% paraformaldehyde for analysis by flow cytometry.

### HIV uptake assay

AT-2 inactivated HIV BaL was incubated in the presence of SP-D (5 µg/mL) in R10 made 5 mM CaCl_2_ for 1 h at 37 °C. Collectins complexed with HIV were then added to iMDDCs (1×10^5^) aliquoted into wells of a 96-well tissue culture treated microtiter plate and incubated for 2 h at 37 °C. Cells were pelleted and extensively washed with RPMI and with PBS containing 10 mM EDTA to remove externally bound HIV particles. DCs incubated with AT-2 inactivated HIV BaL were lysed in PBS with 1% (v/v) Triton X-100 and viral uptake was measured by p24 ELISA according to the manufacturer's instructions (Immunodiagnostics).

### DC-mediated HIV transmission assay

DC-mediated HIV transmission assays were performed as described previously (19). Infectious HIV BaL particles were incubated in the presence of SP-D (1–10 µg/mL) for 1 h at 37 °C before incubation with iMDDCs (1×10^5^) for 2 h at 37 °C. Cells were extensively washed to remove unbound virus, and then placed in a fresh 96-well plate for co-culture with PM1 cells (1×10^5^/well). Culture supernatants were collected after 4–5 days and virus replication was monitored by p24 ELISA.

### Statistical analysis

Statistical comparisons were made using the unpaired t-test with Welch's correction and for more than two groups one way ANOVA with one control group was used. Means, standard derivations and values of p for the differences between means were calculated using Excel software (Microsoft) or GraphPad Prism (GraphPad Software) version 5. Values p<0.05 was considered statistically significant.

## Results

### Binding of SP-D to HIV by ELISA and Surface Plasmon Resonance

**Figure 1:**
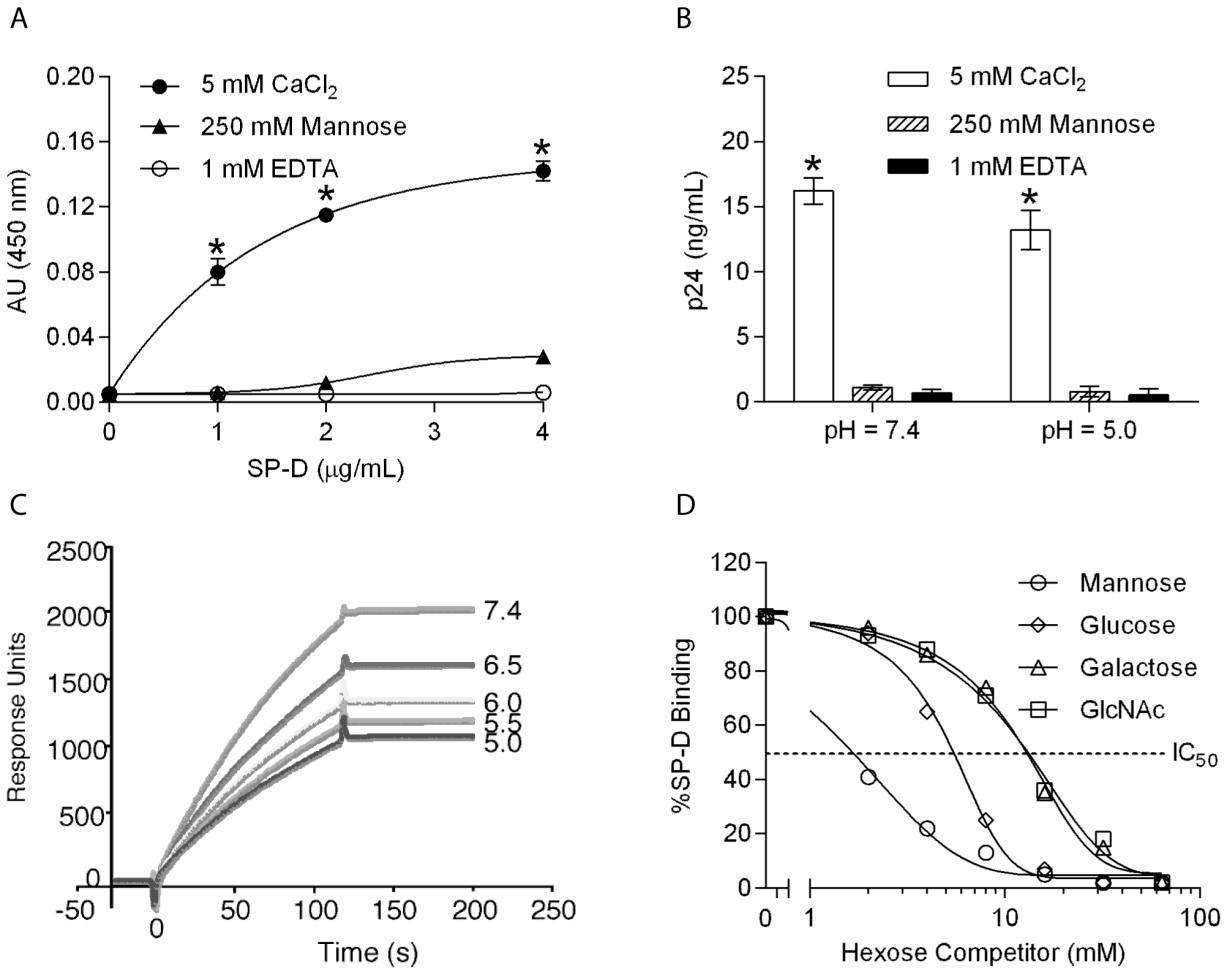
SP-D binds to HIV. **A**: Binding of different concentrations of native SP-D to immobilized AT-2 inactivated HIV BaL (10 µg/mL). SP-D specifically binds to HIV in the presence of 5 mM CaCl_2_ (•), but not in the presence of 250 mM mannose (▴) or 1 mM EDTA (◯). Each data point represents the mean ± S.D. (n = 3). * shows statistical significant increase in the binding of SP-D to HIV in the presence of calcium compared to the presence of mannose or EDTA (p <0.05). **B**: Binding of inactivated HIV BaL (10 µg/mL) to immobilized SP-D (2 µg/mL) at pH 7.4 and 5.0, as estimated by the release of p24 antigen by lysis of HIV particles. SP-D binds to HIV in the presence of 5 mM CaCl_2_ (white), but binding is reduced in the presence of 250 mM mannose (striped) or 1 mM EDTA (black). Each data point represents the mean ± S.D. (n = 3). **C**: Surface plasmon resonance response showing the binding of HIV BaL particles to immobilized SP-D at incremental pH values from pH 5.0 to 7.4. Competition of SP-D binding to inactivated HIV by hexoses. **D**: Competition of HIV BaL particles (10 mg/mL) binding to immobilized SP-D by various hexoses. The IC_50_ values for SP-D were as follows: mannose (1.5 mM)>glucose (5.4 mM) >>GlcNAc (12.2 mM = D-galactose (12.2 mM). Dotted line shows IC_50_.

To determine whether SP-D can bind to HIV, we immobilized a fixed amount (10 µg/mL) of AT-2 inactivated HIV BaL particles in a microtiter plate. Inactivation with AT-2 has previously been shown to have no effect on the conformational structure of HIV particles in comparison to infectious virus [39]. Serial dilutions of SP-D (0–4 µg/mL) were allowed to bind to the immobilized HIV BaL particles and the bound collectin was detected using an anti-SP-D (n/CRD) antibody. SP-D bound to immobilized BaL HIV particles in the presence of CaCl_2_, but the binding was inhibited by both mannose and EDTA, suggesting involvement of the C-type lectin CRD (Figure 1A). Given our interest in examining the role of SP-D in early phase HIV infection, we investigated whether lowering the pH to the physiological buffering range of the vaginal tract (approximately pH 5.0; [40]) could affect the binding of SP-D to virus using the ELISA. Incubation of AT-2 inactivated HIV BaL in microtiter wells coated with SP-D further verified the interaction between the collectins and HIV. The binding was studied at both a pH of 7.4 and 5.0 in order to replicate the environments of the body's blood pH value and the pH of the female vaginal tract, respectively. Bound viral particles were lysed in PBS with 1% (v/v) Triton X-100 and p24 antigen was measured by ELISA. Immobilized SP-D binds to HIV BaL particles at a pH of 7.4 in the presence of calcium, but is inhibited by mannose and EDTA. At a pH of 5.0, binding of SP-D to particles to immobilized SP-D was significant in the presence of CaCl_2_, but was inhibited by mannose and EDTA (Figure 1B). Similar results were obtained for the binding of SP-D to HIV MN particles at a pH of 7.4 (not shown). To verify the interaction between SP-D and HIV using another methodology, we further investigated the nature of the SP-D binding to HIV by surface plasmon resonance. SP-D was immobilized on a BIACore chip, as we have previously described for SP-A [28]. The binding was dependent on the presence of calcium as no binding was seen in the presence of EDTA. Surface plasmon resonance analyses confirmed the interaction between SP-D and HIV and also revealed a relative difference in the affinity of SP-D that is pH-dependent (Figure 1C). Association and dissociation constants were calculated using the approach described by MacKenzie et al [36]. Interestingly, while the dissociation constants of SP-D were essentially unchanged by pH, the association constants were notably different, with a calculated relative binding affinity of 1.68 at a pH of 7.4 normalised to the binding affinity at pH 5.0, which we set to 1.0 (relative binding affinity at pH 6.5 = 1.37, pH 6.0 = 1.21, pH 5.5 = 1.08). The absolute affinity of SP-D for HIV could not be calculated given that the molecule is a tetramer of individual SP-D trimers, which is why we have calculated and report only the relative affinities.

We then performed the binding assay at pH 7.4 in the presence of increasing concentrations of hexoses (0–40 mM). D-mannose was particularly effective at inhibiting the SP-D-HIV interaction relative to the other hexoses (Figure 1D). Glucose also effectively inhibited the interaction of both collectins to HIV in comparison to GlcNAc and D-galactose (Figure 1D). These results further support our ELISA results that SP-D binds to HIV through the C-type lectin activity of the CRDs.

### Binding of SP-D to the gp120 envelope protein of HIV

**Figure 2:**
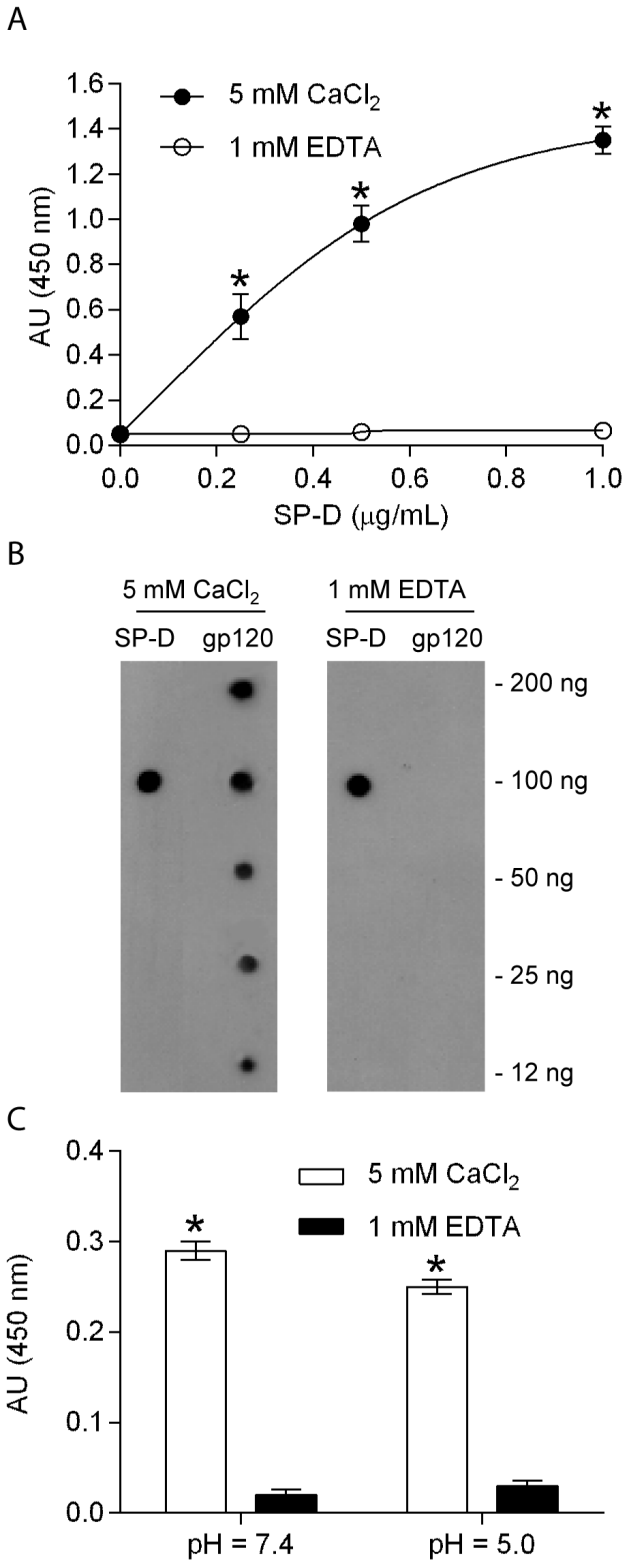
SP-D binds to recombinant gp120. **A**: ELISA assay showing the binding of different concentrations of native SP-D to immobilized recombinant gp120 IIIB (2 µg/mL) made in CHO cells. SP-D specifically binds to gp120 in the presence of 5 mM CaCl_2_ (•), but not in the presence of 1 mM EDTA (◯). Each data point represents the mean ± S.D. (n = 3). * shows statistical significant increase in the binding of SP-D to HIV in the presence of calcium compared to the presence of mannose or EDTA (p <0.05). **B**: Dot blots showing the binding of SP-D to gp120. SP-D (100 ng) or the indicated amounts of recombinant gp120 were dotted on a nitrocellulose membrane, and the blots were incubated with SP-D (200 ng/mL) in either 5 mM CaCl_2_ or 1 mM EDTA. SP-D on the blot was detected by anti-SP-D (n/CRD) antibody. The antibody detected the positive SP-D control in both SP-D lanes. SP-D binds specifically to gp120 on the blots in a concentration-dependent manner in the presence of calcium but not in the presence of EDTA (gp120 lanes). **C**: Binding of gp120 IIIB (2 µg/mL) to immobilized SP-D (2 µg/mL) at pH 7.4 and 5.0. SP-D bound to HIV in the presence of 5 mM CaCl_2_ (white), but binding was reduced in the presence of 1 mM EDTA (black). Each data point represents the mean ± S.D. (n = 3). * shows statistical significant increase in the binding of SP-D to HIV in the presence of calcium compared to the presence of EDTA (p <0.05).

We have previously shown that SP-A interacts with gp120 [28]. To directly determine whether SP-D can bind to gp120, we immobilized a fixed amount (2 µg/mL) of recombinant gp120 IIIB expressed in CHO cells on an ELISA plate. SP-D (0–1 µg/mL) was allowed to bind to gp120 IIIB, and the bound protein was detected by biotinylated anti-SP-D (n/CRD) antibody. The result showed that SP-D binds to gp120 IIIB in a concentration- and calcium- dependent manner (Figure 2A) similarly to the binding of inactivated HIV BaL particles. The binding was further confirmed by dot blot assays where SP-D showed a binding to immobilized recombinant gp120 in the presence of calcium whereas no binding was seen in the presence of EDTA (Figure 2B). To characterize the binding between SP-D and gp120 in more detail we examined the binding at pH 7.4 and pH 5.0. SP-D bound to gp120 at both pH 7.4 and 5.0 in the presence of calcium whereas no binding was seen in the presence of EDTA (Figure 2C).

### Sugar competition of SP-D binding to recombinant gp120

**Figure 3:**
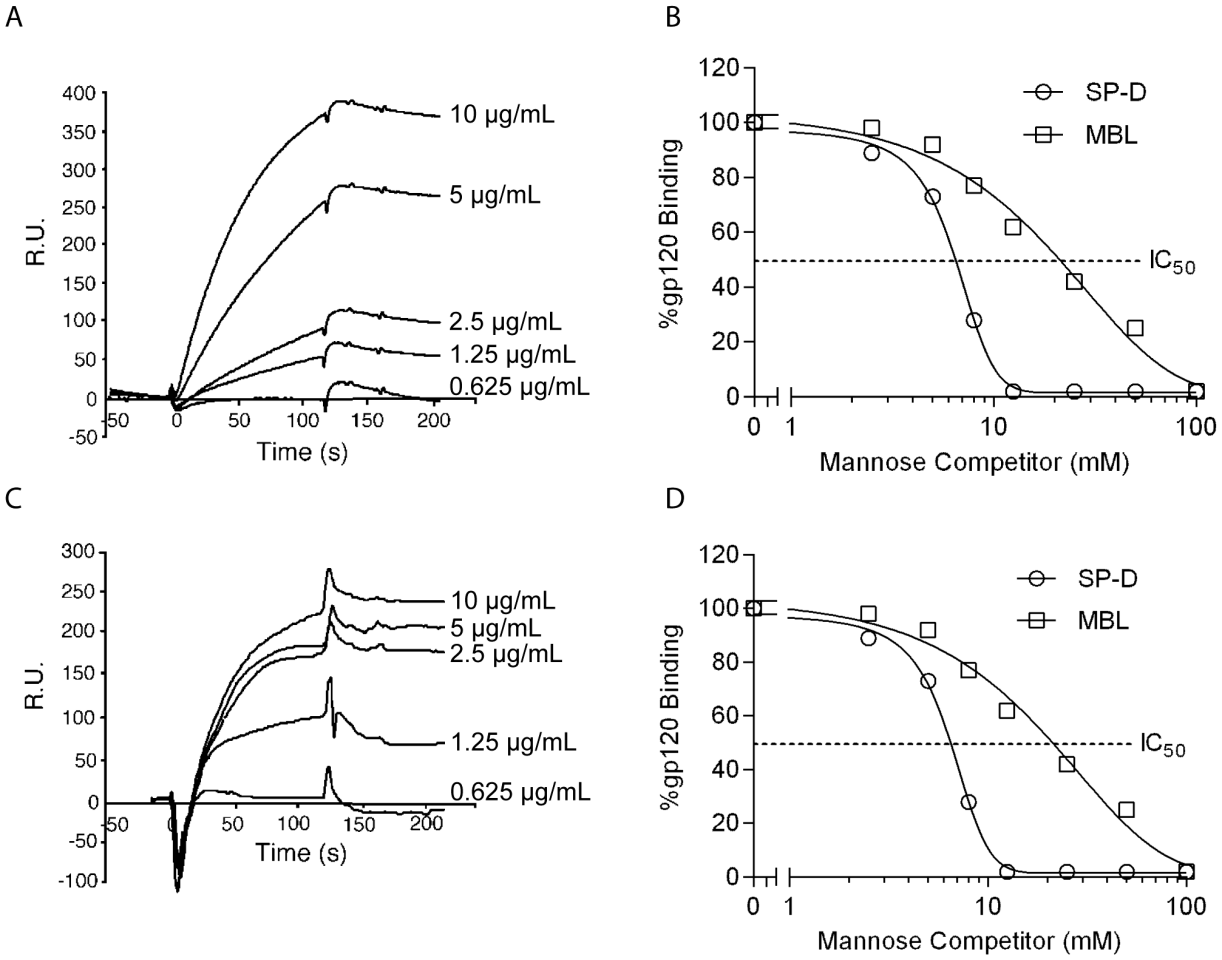
Competition of SP-D binding to gp120 by hexoses. **A**: Surface plasmon resonance response showing the binding of SP-D to biotinylated recombinant gp120 IIIB immobilized on a streptavidin chip. Each trace indicates the response units recorded at a given concentration of SP-D (10, 5, 2.5, 1.25, and 0.625 µg/mL) in 5 mM CaCl_2_-containing (HSC) buffer (traces are in order of the highest to lowest concentration). **B**: Competition of SP-D (5 µg/mL) binding to gp120 with hexoses. The effect of different concentrations of hexoses in competing with gp120 for SP-D binding. The IC_50_ values were as follows: maltose; 6.5 mM>mannose; 7.8 mM>>GlcNAc; 22.9 mM>D-galactose; 32.2 mM. Dotted line shows IC_50_. **C**: Competition of SP-D binding to mannan with gp120. The effect of different concentrations of gp120 (0, 1.625, 3.125, 6.25, and 12.5 µg/mL) on the binding of SP-D (5 µg/mL) to mannan was determined by the changes in the response units. An increase in the gp120 concentration results in a decrease in the surface plasmon resonance response. **D**: Competition of gp120 binding for SP-D and MBL to mannan. Dotted line shows IC_50_

To further characterize the interaction between gp120 and SP-D with greater sensitivity, we examined the binding of SP-D to biotinylated gp120 IIIB immobilized on a BIACore chip. The surface plasmon resonance (SPR) showed that SP-D (0–5 µg/mL) bound to gp120 in a concentration-dependent manner under physiological salt conditions (Figure 3A). To investigate the nature of SP-D binding to gp120 further, we allowed SP-D (5 µg/mL) to bind to gp120 IIIB immobilized on a BIACore chip in the presence of increasing concentrations of hexoses (0–40 mM). BIACore assays behaved similarly to a conventional ELISA system with maltose and D-mannose inhibiting the interaction between SP-D and gp120 with relatively low values compared to N-acetyl glucosamine (GlcNAc) and D-galactose (Figure 3B). These results suggest that SP-D binds to gp120 through its carbohydrate recognition domain, while the strong inhibition by D-mannose suggests that the high mannose glycans on gp120 are potential ligands for SP-D. To further explore the interaction between SP-D and gp120, we allowed SP-D (5 µg/mL) to bind to immobilized mannan on a BIACore chip in the presence of increasing concentrations of gp120 (0–100 µg/mL) and this showed a concentration dependent inhibition of the interaction (Figure 3C). MBL has been reported to bind to HIV and gp120 [26], [41] and we wanted to compare the binding of SP-D to gp120 with the binding of MBL to gp120. Inhibition of SP-D binding to mannan occurred at a significantly lower concentration of gp120 than for the serum collectin MBL. 50% inhibition was achieved at a 5 µg/mL concentration of gp120 for SP-D and at a 15 µg/mL concentration for MBL (Figure 3D).

### Competition of gp120 binding proteins to gp120 and SP-D

**Figure 4:**
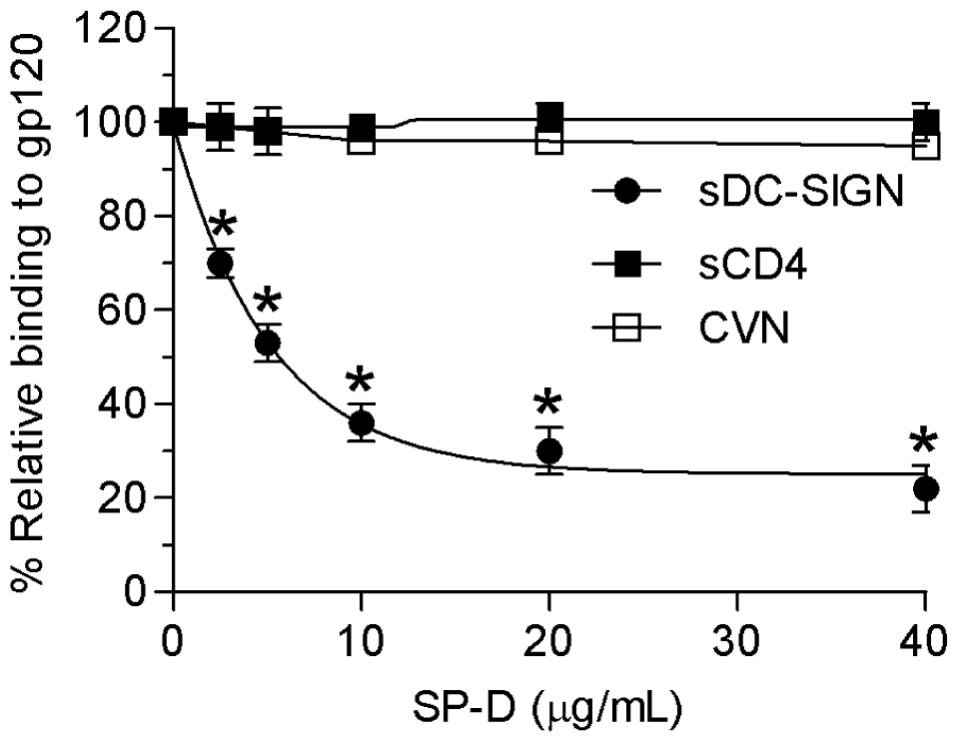
Competition of gp120 binding to gp120 binding proteins by SP-D. A fixed amount of recombinant gp120 IIIB (2 µg/mL) was immobilized on an ELISA plate, and, soluble DC-SIGN (•) or soluble CD4 (▪) was allowed to bind in the presence of the indicated concentrations of SP-D (0–40 µg/mL). Absorbance (450 nm) in the absence of competitor was considered as 100% binding. Each data point represents the mean ± S.D. (n = 3).

To further define the specific SP-D epitope on gp120, we evaluated the effect of SP-D on the binding of several gp120 binding proteins to monomeric gp120 IIIB (Figure 4). A fixed amount of recombinant monomeric gp120 was immobilized on an ELISA plate as monomeric gp120 is found in concentrations of 12–92 ng/mL in HIV-1 infected serum [42]. Soluble biotinylated CD4 or DC-SIGN were then allowed to compete for the binding to gp120 in the presence of increasing concentration of SP-D (0–40 µg/mL). We also chose to determine whether SP-D could compete the binding of the neutralizing protein cyanovirin (CVN), which binds to mannose-dependent epitopes on gp120 [43]. The binding of sCD4 and CVN was not affected by the presence of SP-D at all SP-D concentrations tested, indicating that the binding sites for sCD4 and CVN do not overlap with SP-D (Figure 4). The binding of sDC-SIGN was statistically significant (p<0.05) inhibited at all SP-D concentrations tested with a maximum inhibition of 80% at the highest SP-D concentration tested when compared to no SP-D present indicating an overlap with the binding site for SP-D (Figure 4).

### SP-D agglutinates HIV and inhibits infectivity of PM1 cells with HIV

**Figure 5:**
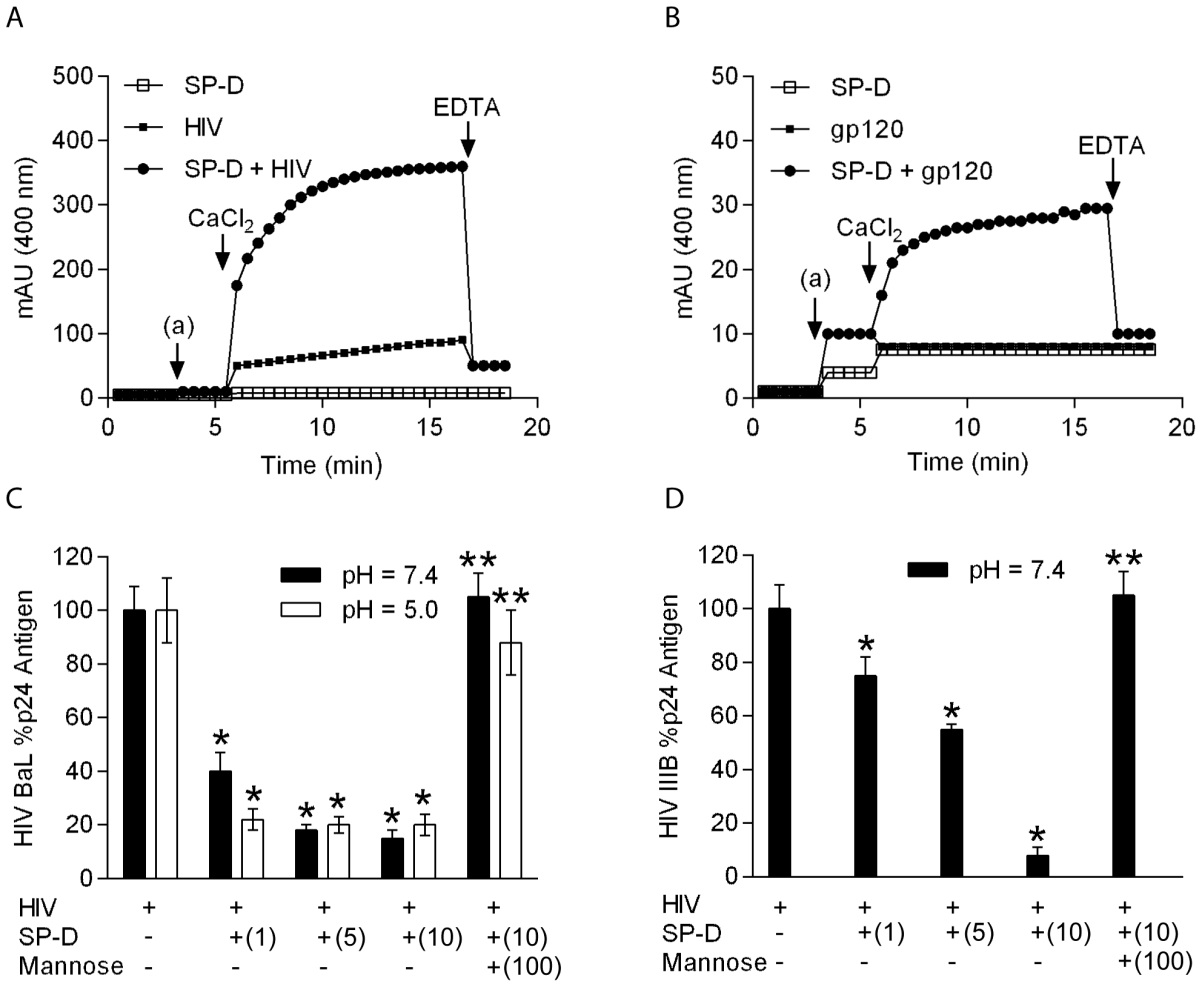
SP-D agglutinates and inhibits infectivity of HIV. **A**: Kinetics of HIV particle agglutination by SP-D. SP-D (10 µg/mL) (□), HIV BaL (10 µg/mL) (▪), or SP-D and HIV BaL (•) were added at point (a) after initial equilibrium at 20 mM Tris-HCl, 150 mM NaCl. The turbidity change at 400 nm was monitored at 30 s intervals. After 3 min stabilization, 5 mM CaCl_2_ (final concentration) was added to the sample cuvette and turbidity changes were measured again. Addition of EDTA (10 mM, final concentration) resulted in disassociation of SP-D induced aggregates. The same experimental approach was used for **B**: SP-D (10 µg/mL) (□), gp120 IIIB (10 µg/mL) (▪), or SP-D and gp120 (•). The results of a representative experiment from three separate experiments for each assay are shown. **C**: Infectious HIV BaL particles were preincubated at a pH of 7.4 (black) or 5.0 (white) in the presence and absence of the indicated concentrations of SP-D (1–10 µg/mL) before inoculation with PM1 cells. The PM1 cells were washed and day 5 culture supernatants were analyzed by p24 ELISA. Numbers in parenthesizes are the SP-D concentration in µg/mL and the mannose concentration in mM. **D**: Infectious HIV IIIB particles at pH 7.4. Each data point represents the mean ± S.D. (n = 3). * shows statistically significant decrease in p24 antigen in the presence of SP-D compared to no SP-D present(p<0.05). **shows statistically significant increase in p24 antigen in the presence of mannose relative to treatment with SP-D alone (p<0.05). Numbers in parenthesizes are the SP-D concentration in µg/mL and the mannose concentration in mM.

One of the important clearing mechanisms of SP-D is thought to be agglutination of virus which has been shown for influenza A virus [44]. In order to see if SP-D was capable of agglutinating HIV, SP-D was incubated together with virus and the turbidity change at 400 nm over time was measured in the presence of calcium and then later after the addition of EDTA. SP-D agglutinated HIV BaL particles in the presence of calcium and the agglutinated complexes disassociated by the addition of EDTA (Figure 5A). SP-D or virus alone did not self-agglutinate in the presence of calcium (Figure 5A). A similar effect was seen when SP-D was incubated with gp120 from HIV IIIB in the presence of calcium and the complexes would disassociate by the addition of EDTA (Figure 5B).

As our studies showed that SP-D interacts with both HIV and gp120 and agglutinates both HIV and gp120, we next asked whether SP-D could inhibit HIV infectivity. Infectious strains of HIV BaL and IIIB were incubated with increasing concentrations of SP-D before incubating with PM1 or C8166 cells. Experiments with HIV BaL were performed at both pH 7.4 and 5.0 as R5 strains, such as HIV BaL, are typically found in the early phase infection whereas experiments with HIV IIIB (which is an X4 strain and is mainly found during late-phase infections) was only performed at pH 7.4. The pH dependent incubation was conducted during the initial incubation of HIV particles with SP-D and then the SP-D-HIV complexes were incubated with PM1 cells. All cells were washed post-infection and then resuspended in pre-warmed medium at a pH 7.4. There was a SP-D concentration dependent inhibition of HIV BaL and at the highest concentration of 10 µg/mL, SP-D inhibited infectivity of HIV BaL to around 15% at pH 7.4 and 20% at pH 5.0 (Figure 5C). A similar SP-D concentration dependent infectivity was seen for HIV IIIB particles at pH 7.4 where SP-D at the highest concentration of 10 µg/mL tested reduced the infectivity to 5% when compared to no SP-D present (Figure 5D). The effect was reproducible with different preparations of SP-D and the inhibitory effect of SP-D was completely abrogated when the pre-incubation of SP-D with HIV particles was performed in the presence of 100 mM D-mannose for both HIV BaL and IIIB strains.

### SP-D enhances the binding of gp120 to iMDDCs and enhances viral uptake and transfer of HIV from iMDDCs to CD4+ T cells

**Figure 6:**
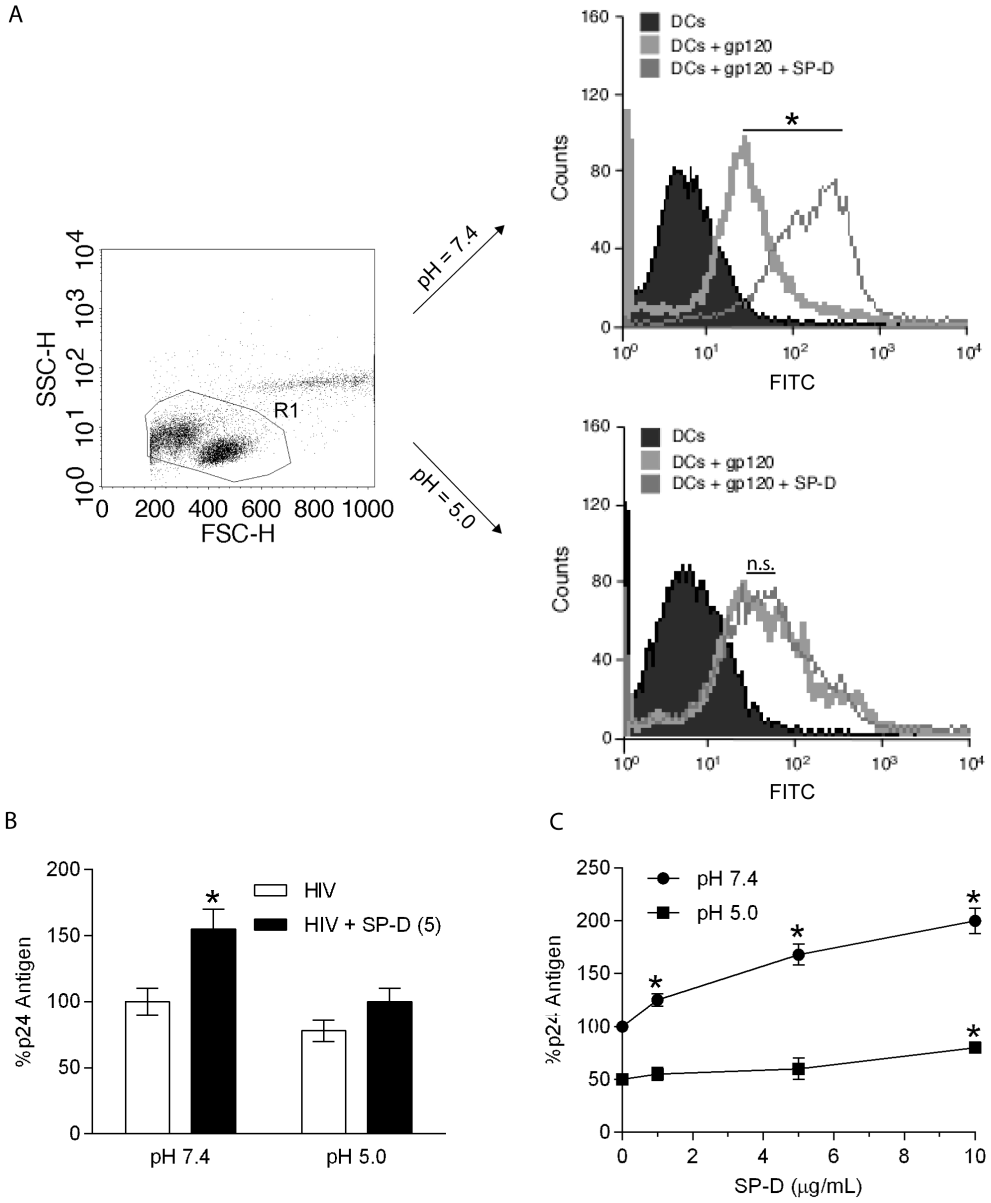
SP-D enhances binding of gp120 to iMDDC and enhances HIV capture and transfer by iMDDCs. Binding of FITC-labeled gp120 to iMDDCs in the presence and absence of SP-D (5 µg/mL) at **A**: pH of 7.4 or 5.0. DCs were incubated with FITC-labeled gp120, or FITC-gp120 and SP-D at 4 °C for 1 h and then washed extensively before analysis by flow cytometry. The iMDDCs were initially analysed in FACS dot plot showing size (FSC-H) and granularity (SSC-H) characteristics. A gate (R1) was used to select for immature DCs and these were further analysed for binding of the FITC labeled gp120 or FITC labeled gp120 in the presence of SP-D. A geometric mean fluorescence intensity (GMFI) was calculated for each histogram plot and statistical analysis was performed using an unpaired t-test with Welch's correction. * shows statistically significant increase in p24 antigen uptake by DCs in the presence of SP-D compared to the uptake with no SP-D present (p <0.05). N.s indicates no statistically significance between gp120 and the presence of SP-D and no SP-D present (p = 0.409). **B**: Virus captured by iMDDCs in the presence or absence of SP-D (5 µg/mL). DCs were incubated with AT-2 inactivated HIV BaL particles for 2 h at 37 °C, washed extensively in 10 mM EDTA containing buffer, and lysed for analysis by p24 ELISA. The value of virus captured by iMDDCs in the absence of SP-D at pH 7.4 was defined as 100%. Each bar represents the mean ± S.D. (n = 3). *shows statistically significant increase in p24 antigen uptake by DCs in the presence of SP-D compared to no SP-D present (p <0.05). **C**: Infectious HIV BaL was incubated with iMDDCs in the presence or absence of the indicated concentrations of SP-D (0–10 µg/mL) at a pH of 7.4 or 5.0. Unbound virus was removed by washing and iMDDCs were then co-cultured with PM1 cells for five days before levels of p24 antigen in the culture supernatants was measured by ELISA. The value of virus transferred by iMDDCs to PMI cells in the absence of SP-D at pH 7.4 was defined as 100%. *shows statistically significant increase in p24 antigen uptake by DCs in the presence of SP-D compared to no SP-D present (p <0.05).

We have previously shown that SP-A enhances the binding of gp120 to iMDDCs [28]. We were interested to know if SP-D had the same effect on gp120 and iMDDCs and examined how SP-D affected the interaction between gp120 and IMDDCs. FITC-labeled gp120 (2 µg/mL) was incubated with iMDDCs in the presence or absence of SP-D at both pH 7.4 and 5.0. Experiments were performed at 4 °C to prevent internalization of gp120 by iMDDCs. Based on the geometric mean fluorescence intensity (GMFI) SP-D enhanced the association of FITC-labeled gp120 to iMDDCs at pH 7.4 when compared to iMDDCs incubated with only gp120 in the absence of SP-D (Figure 6A, p<0.05). However, at pH 5.0 the increase seen in associated gp120 in the presence of SP-D at pH 7.4 was much less and there was no statistical difference between the association with gp120 and iMDDCs in the presence or absence of SP-D (Figure 6A, p = 0.409).

As several reports have shown that SP-D enhances the phagocytosis of a number of microbial pathogens [5], [15], [45], we investigated whether SP-D could enhance the uptake of HIV particles by iMDDCs. AT-2 inactivated HIV BaL particles (10 µg/mL) were incubated with iMDDCs in the presence and absence of SP-D (5 µg/mL) at a pH of both 7.4 and 5.0 at 37 °C, with modulation of the pH carried out in the same manner as the gp120-DC binding experiment. After incubation, cells were washed in 10 mM EDTA-containing buffer and then lysed and analyzed for p24 antigen content. SP-D significantly enhanced the uptake of particles by approximately 50% at a pH of 7.4 (p<0.05), but although SP-D did increase the uptake of HIV at pH 5.0 this was not statistically significant (Figure 6B). Thus, the results from the uptake experiment corroborate the results from our gp120-iMDDC binding experiment, and collectively suggest that SP-D enhances the interaction and phagocytosis of HIV by DCs.

To investigate whether SP-D affects DC-mediated transfer of HIV to CD4+ T cells, iMDDCs were incubated with infectious HIV BaL particles in the presence and absence of SP-D (1-10 µg/mL) at both a pH of 7.4 and a pH of 5.0. Modulation of the pH was carried out in the same manner as the gp120-iMDDCs binding and HIV uptake experiments. After the incubation, iMDDCs were washed extensively and then co-cultured with PM1 cells for 5 days before analysis of culture supernatants by p24 ELISA. SP-D enhanced iMDDC-mediated transfer of HIV at a pH of 7.4 in a dose-dependent manner, with an approximately 2-fold statistically significant enhancement of infection at the highest SP-D concentration of 10 µg/mL tested (Figure 6C, p<0.05). SP-D also enhanced the transfer of HIV at a pH of 5.0 by approximately 50% relative to the DC control at the same pH and this was also a statistically significant increase (Figure 6C, p<0.05). Thus, these results suggest that while SP-D protects CD4+ cells from direct infection, it may also have a harmful effect on the body's defence against HIV in both the lungs and the vaginal tract via interactions with DCs.

## Discussion

In this study, we investigated the interaction of SP-D with several strains of HIV. We have previously shown that SP-A interacts with HIV and enhances dendritic cell-mediated viral transfer [28]. SP-D has also previously been shown to interact with HIV and gp120 and inhibit HIV replication in *in vitro* cell culture assays [29]. Therefore, we sought to characterize the interaction between SP-D and HIV in more detail and to investigate whether this interaction could have a significant effect on HIV pathogenesis as found for SP-A.

The results in this report clearly show that SP-D binds to intact AT-2 inactivated HIV particles in a calcium dependent manner that is inhibitable by mannose and EDTA (Figure 1A). Infectious HIV particles inactivated by AT-2 have previously been shown to retain the conformational and functional integrity of the surface proteins [39] indicating that SP-D would bind in a similar manner to these particles. Furthermore, competition assays with different hexoses using the surface plasmon resonance technique showed that mannose had the lowest IC_50_ value (1.5 mM) followed by glucose (5.4 mM) and then galactose and GlcNAc (both 12.2 mM) (Figure 1B). The order of these saccharide selectivities is in the same order as Crouch and colleagues previously have shown for natural and full-length recombinant human SP-D [46]. The requirement of calcium, the binding being inhibited by EDTA and mannose, and the saccharide specificity in competition assays suggests that binding of SP-D to HIV is mediated by the lectin binding site in the CRD domain and that SP-D would interact with a glycoconjugate on HIV. As one of the important ways of viral transmission is via the female urogenital tract, which has a pH value around 5, the interaction between SP-D and HIV was investigated at both pH 5.0 and 7.4 (Figure 1B and 1C). This showed that SP-D can bind to HIV at both pH values but that binding at pH 5.0 results in a lower association constant and thus relative affinity, when compared to interaction between SP-D and HIV at pH 7.4. The binding profile of SP-D in the pH range from 7.4 to 5.0 is similar to what we have previously reported for SP-A but in contrast to DC-SIGN, which loses its binding capacity to HIV at pH 5.0 [28]. However, although SP-A showed an increased binding to HIV at pH 5.0 when compared to pH 7.4 in the presence of EDTA [28] no binding was seen to HIV with SP-D in the presence of EDTA at both pH values indicating that the interaction is a mainly a calcium dependent sugar-lectin interaction. SP-D has previously been shown to interact calcium-dependently with gp120 [29] and the interaction was dependent on the presence of glycans on gp120 as no binding was observed to de-glycosylated SP-D [28]. The results in this paper also showed a calcium-dependent interaction between SP-D and gp120 and furthermore, we show that the interaction occurred at both pH 7.4 and 5.0 (Figure 2) consistent with what we observed for whole inactivated HIV particles (Figure 1). In order to further characterize the interaction between SP-D and gp120 we performed competition assays with saccharide hexoses. Maltose was found to be the best inhibitor with an IC_50_ value of 6.5 mM followed by mannose (7.8 mM), GlcNac (22.9 mM) and D-galactose (32.5 mM) (Figure 3B). The relative order of these hexoses is in accordance with the IC_50_ values we found for competition assays between SP-D and whole inactivated HIV particles (Figure 1D) and what has been observed for whole SP-D molecules and hexoses [46]. This was confirmed by the finding that SP-D has a stronger affinity for gp120 than seen for MBL (Figure 3D) which is in accordance with the report by Hartshorn and colleagues showing that SP-D inhibited infectivity of HIV and influenza virus *in vitro* at significantly lower concentrations than MBL [28], [47].

**Figure 7:**
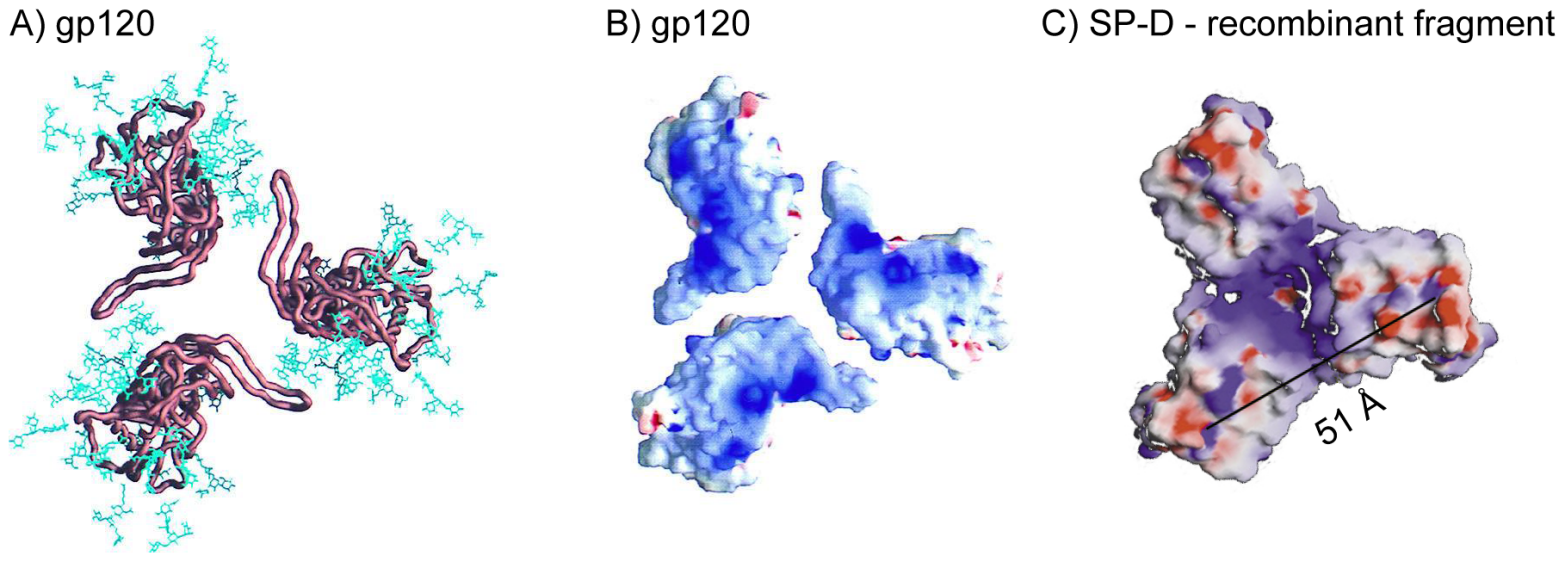
Computer generated model of trimeric gp120 and human SP-D. **A**: Cα worm representations of core gp120 (copper brown) and the gp120 carbohydrate cores (blue), the (*N*-acetylglucosamine)_2_-(mannose)_3_cores shared by both high-mannose and complex N-linked glycan moieties. The carbohydrate shown here represents approximately half the carbohydrate on gp120, with the rest extending further from the gp120 surface. Distance from glycan tip to glycan tip is estimated to be 110 Å. View from target cell membrane. **B**: The electrostatic surface of gp120 for the core. The electrostatic potential is depicted at the solvent-accessible surface, which is colored according to the local electrostatic potential, regions of positive potential are shown in blue and negative potential is in red. View from target cell membrane. **C**: The electrostatic surface potential of the recombinant fragment of hSP-D. The molecule is shown looking directly on top of the CRDs. Regions of positive potential are shown in blue and negative potential is in red. The distance between two carbohydrate binding calcium ions is 51 Å and indicated on the figure. A and B from Kwong et al [48] with permission. C modified from Hakansson et al [49]with permission. B and C visualized using the program GRASP [64].

To further characterize the interaction between SP-D and gp120 we performed an ELISA based inhibition assay to test the ability of SP-D to interfere with the binding of known ligands for gp120. As shown in Figure 4, these proteins can be divided into two groups: 1) CVN and sCD4 did not alter the SP-D binding profile to immobilized gp120 and 2) DC-SIGN inhibited the binding. Computer based modelling of a trimeric gp120 with glycans [48] show a remarkable complementarity to the crystal structure of a recombinant fragment of human SP-D [49]. These molecules are not only complementary in terms of oligomerized shape but also with electrostatic charge and the location of glycans on gp120 and the carbohydrate binding sites in human SP-D (Figure 7). However, the distance across the computer generated oligomeric model of gp120 was estimated to be approximately 110 Å from tip of a glycan to tip of another glycan across the oligomerised molecule whereas the distance between the binding sites in SP-D has been calculated to be 51 Å. This opens up the possibility that SP-D could bind to the center of the oligomerized gp120 molecule, by binding to glycans located in the V3 loop, and thereby there would still be space for some of the other ligands investigated here also targeting high mannose structures on gp120. As the recombinant protein CVN neutralises HIV by targeting a specific mannose-dependent epitope on gp120 [43] the observation here with the differential results shows that the SP-D and CVN proteins probably target different epitopes on gp120. The computer generated modelling of trimerized gp120 showed that the CD4 binding site is predominantly located on the side of the trimerized molecule and distant from the glycans which is predominantly located on top of the trimerized molecule (Figure 7). The competition assay showed that SP-D did not affect the interaction of sCD4 with gp120. This result was confirmed by immobilizing sCD4 and testing for binding to whole inactivated HIV particles or gp120 in the presence of SP-D. Both HIV and gp120 showed no change in the binding to sCD4 in the presence of SP-D (data not shown). This confirms the suggestion that SP-D binds to the glycans found on the top of the trimerized gp120 molecule and therefore does not interfere with the CD4 binding site located on the side of the trimerized molecule. Given that DC-SIGN has been shown to target the high mannose oligosaccharides on gp120 [50], it was expected that SP-D would compete this interaction. Only DC-SIGN was found to have decreased binding to gp120 in the presence of SP-D indicating that the epitopes on gp120 that SP-D and DC-SIGN bind to may overlap. This decreased binding of DC-SIGN to gp120 in the presence of SP-D was confirmed by immobilizing DC-SIGN and testing for binding of whole inactivated HIV particles or gp120 in the presence of SP-D. Both HIV and gp120 showed decreased binding in the presence of SP-D (data not shown). These results are different from our previous findings with SP-A, which inhibits the binding of both sCD4 and DC-SIGN to gp120 [28]. As with the result for CVN binding, these results indicate that SP-A and SP-D bind to gp120 in different ways.

The concentration of SP-D in bronchoalveolar lavages from healthy volunteers ranges from 100–900 ng/mL [51], [52]. As SP-D is diluted during the procedure of BAL the concentration of SP-D *in situ* in the lung would be higher and our experiments performed here are probably in that concentration range. SP-D was found to agglutinate both gp120 and intact inactivated HIV BaL particles in the presence of calcium (Figure 5). This is the first time that a collectin has been shown to agglutinate HIV. SP-D was more potent in agglutinating whole HIV particles than gp120 molecules, which is not surprisingly given the size of HIV particles compared to gp120 molecules and the number of gp120 molecules found on each HIV particles. The ability of SP-D to agglutinate influenza A virus has been shown to be an important factor for protection against the virus [44] and we assume that a similar protective function could be possible for SP-D and HIV. The agglutinating function of SP-D towards HIV is not unique as we have also found that SP-A was cable of agglutinating HIV particles under the same experimental conditions (data now shown).

We have shown that SP-D inhibited infectivity of PM1 cells with the R5 strain HIV BaL and C8166 cells with the X4 strain HIV IIIB (Fig. 5). Assays with HIV BaL were performed at a pH value of 7.4 and 5.0, representative of the overall body and the vaginal tract, respectively, as R5 strains are typically found in early phase infection [53]. Experiments with HIV IIIB were performed at a pH of 7.4, as particles are found in the lungs during late-term infection usually have a CXCR4 receptor tropism [53]. SP-D was found cable of inhibiting both HIV strains to a degree of only 5–10% infectivity at pH 7.4 for both HIV strains tested and to approximately 20% at pH 5.0. Thus, these results indicate that SP-D may have a significant effect *in vivo* by protecting CD4+ cells from direct infection in a variety of physiological environments.

We show here that SP-D enhanced the association of FITC labeled gp120 with iMDDCs at pH 7.4 (Figure 6A) indicating that SP-D would facilitate the association between HIV and iMDDCs. This was performed at 4 °C assuming that this would result in association only and no active uptake would take place. In order to be able to differentiate between association and uptake, these results should be verified in the future using a quenching step and/or confocal microscopy) fluorescence. That SP-D facilitated the association between HIV and iMDDCs was seen when using HIV BaL at both pH values of 7.4 and 5.0 (Figure 6C). The enhancement of DC-mediated transfer of infection (Figure 6D) suggests that SP-D would likely facilitate HIV infection through this alternate viral dissemination pathway *in vivo*. The localization of cells expressing SP-D in the gastrointestinal mucosa and the female vaginal tract places the collectin at an important site to affect virus interaction with DCs during early phase infection [21], [23], while the presence of SP-D in the respiratory tract would facilitate the interaction of HIV with lung DCs. The mechanism of SP-D-mediated enhancement of DC viral transfer that we observed most likely involves the increase in gp120 binding and viral uptake by DCs (Figures 5 and 6). Although iMDDCs are a relatively good model for DC subsets involved in HIV infectivity *in vivo* [54] and the levels of p24 Ag are considerably higher with the use of PM1 cells as indicator cells in comparison to DCs alone (data not shown), it is unclear how much viral transfer is attributable to DC trans or cis-infectivity. Experiments with a replication-defective, single cycle reporter HIV would allow for the determination of whether viral replication is necessary for SP-D-mediated enhancement of HIV transfer by DCs.

The decrease in DC-mediated transfer of infectivity as the pH is reduced to 5.0 is likely the result of the loss of activity of HIV-binding cell surface receptors, such as DC-SIGN [28], and impaired HIV infectivity in an acidic environment [55]. The loss of HIV binding by DC-SIGN as the pH was lowered to 5.0 is consistent with previous reports which have shown that a drop in pH results in a conformational change in the DC-SIGN CRD that alters its binding capacity [56]. Our observation that SP-D is still able to bind to HIV as the pH is lowered may therefore account for the amplified effects by the collectin on gp120 binding and HIV uptake at a pH of 5.0 (Figure 6). It is important to remember that in an *in vivo* setting both SP-A and SP-D are present in the lung and the vaginal mucosa and fluid [23], [57] and any HIV particles would probably be interacting with both collectins. This could lead to an enhancement on gp120 binding and HIV uptake and to be further enhanced by the interaction between collectins and the SP-A and SP-D binding molecule DMBT1^gp-340^ [58], [59], which is also present on mucosal surfaces and has also has been shown to bind to HIV and gp120, inhibit infectivity in *in vitro* in cells culture assay and facilitate trans-infection to T-cells [60]–[63], or a number of other cellular candidate receptors for collectins, such as SIRPalpha and calreticulin/CD91 [10].

In summary, this study is the first to establish an interaction between SP-D and HIV at pH values of both 7.4 and 5.0. SP-D also agglutinated HIV particles. Furthermore, our results suggest that SP-D, as we have previously shown for SP-A, may be a dual modulator of HIV infection by protecting CD4+ cells but enhancing the transfer of infection by dendritic cells *in vivo*. Therefore, we believe that our findings for the two collectins SP-A and SP-D as HIV binding factors are important in advancing our understanding of the innate immune response against HIV and how this, in a future setting, could potentially be manipulated into a therapeutic usage against HIV infections.

## Supporting Information

Figure S1 **Characterization of iMDDCs by flow cytometry.** The iMDDCs were initially analysed in FACS dot plot showing size (FSC-H) and granularity (SSC-H) characteristics. A gate (R1) was used to select for immature DCs and these were further analysed for surface markers for cell type and differentiation using FITC labeled antibodies for: CD11c, CD83 and MHC II (HLA-DR). iMDDCs were positive for the DC-specific markers CD11c and HLA-DR, but not for the mature marker CD83. The dot plot and histograms are representative of the FACS screening performed before iMDDCs were used for experimentation.
(TIF)
