# Supplementary figures and images for: Surfactant Protein D Modulates HIV Infection of Both T-Cells and Dendritic Cells
Source: PLoS One. 2013 Mar 18;8(3):e59047. doi: 10.1371/journal.pone.0059047 (PMC3601116; doi:10.1371/journal.pone.0059047)

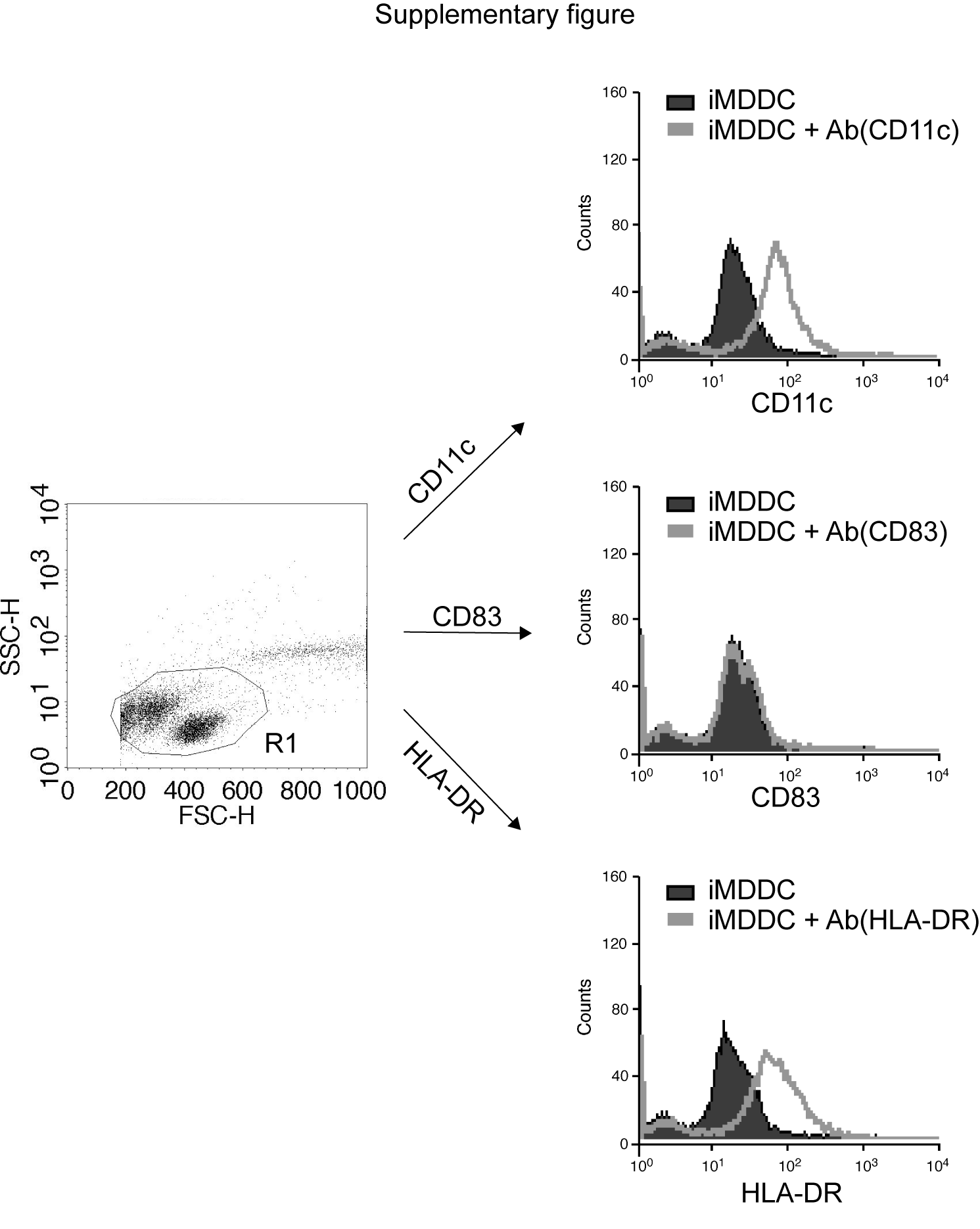

Supplement: Figure S1 — Characterization of iMDDCs by flow cytometry. The iMDDCs were initially analysed in FACS dot plot showing size (FSC-H) and granularity (SSC-H) characteristics. A gate (R1) was used to select for immature DCs and these were further analysed for surface markers for cell type and differentiation using FITC labeled antibodies for: CD11c, CD83 and MHC II (HLA-DR). iMDDCs were positive for the DC-specific markers CD11c and HLA-DR, but not for the mature marker CD83. The dot plot and histograms are representative of the FACS screening performed before iMDDCs were used for experimentation. (TIF) [file pone.0059047.s001.tif]
